# Development of Nanotechnology-Based Drug Delivery Systems for Controlling Clinical Multidrug-Resistant *Staphylococcus aureus* and *Escherichia coli* Associated with Aerobic Vaginitis

**DOI:** 10.3390/pharmaceutics15082133

**Published:** 2023-08-14

**Authors:** Najla Haddaji, Badr Bahloul, Wael Bahia, Olfa Bechambi, Abdelkarim Mahdhi

**Affiliations:** 1 Department of Biology, Faculty of Sciences, University of Ha’il, Ha’il 55436, Saudi Arabia; ol.bechambi@uoh.edu.sa; 2 Laboratory of Analysis, Treatment and Valorization of the Pollutants of the Environment and the Products, Faculty of Pharmacy of Monastir, Monastir 5000, Tunisia; abdelkarim.mahhdhi@fsm.rnu.tn; 3 Pharmaceutical, Pharmacological & Chemical Drug Development Laboratory, Faculty of Pharmacy, University of Monastir, Monastir 5000, Tunisia; badr.bahloul@fphm.u-monastir.tn; 4 Research Unit of Clinical and Molecular Biology (UR17ES29), Department of Biochemistry, Faculty of Pharmacy, University of Monastir, Monastir 5000, Tunisia; bahiawael@gmail.com

**Keywords:** probiotic, biosurfactant, nanotechnology, antibacterial, antibiofilm, vaginosis

## Abstract

The growing prevalence of resistance to antibiotics potentially makes *Escherichia coli* and *Staphylococcus aureus* serious pathogens, necessitating the development of new antimicrobial agents. We extracted crude biosurfactants from a potential probiotic *Bacillus* spp. to control pathogenic bacteria associated with aerobic vaginal infection. Using nanotechnology formulations, we developed nanoemulsions based on biosurfactants at different concentrations (1% and 3.33%). The results showed that these nanoemulsions were stable, with a weighted index of 0.3, and demonstrated broad-spectrum antibacterial activity against *Escherichia coli* and *Staphylococcus aureus*, with MICs ranging between 1.25 and 4 mg/mL. Additionally, the nanoemulsions exhibited interesting antibiofilm effects. All strains became more sensitive to the antibiotics to which they were resistant because of various biosurfactant formulations combined with antibiotics. Lower concentrations of BNE1% and 3.33% were still more efficient than the crude biosurfactants. Our findings demonstrated that the biosurfactant had a strong antibiofilm effect against all tested pathogens. This antibacterial effect can be explained by their ability to alter cell physiology such as cell hydrophobicity and membrane disintegration. Thus, we can conclude that the use of nanotechnology formulations has improved this effect, and the nanoemulsions developed in this study can be used as a potential anti-infectious therapy against multidrug-resistant bacterial strains of clinical origin.

## 1. Introduction

Healthy vaginal microflora primarily consists of Gram-positive bacteria [1]. Nevertheless, pathogenic strains can also be present in small numbers, such as *Gardnerella vaginalis*, *Enterococcus* spp., and *Prevotella* spp., and are not sufficient to cause disease [2]. However, most vaginal infections are caused by *Staphylococcus aureus* and *Escherichia coli* [3]. Another study showed that *Enterococcus* (31%), *Escherichia coli* (21%), and *Streptococcus pyogenes* (22%) were the most prevalent pathogens in vaginal infections [4]. Therefore, during bacterial vaginosis, the vaginal epithelium usually forms biofilms. Then, the formation of protective biofilms plays an important role in the pathogenesis of bacterial vaginosis [5]. Biofilms can develop on almost any surface, whether living or dead [6,7,8]. Biofilms have been suggested to play an important role in the disease process either through preventing the action of antibiotics or through allowing microorganisms to escape host defense mechanisms [9]. It has been suggested that the incomplete eradication of biofilms by antibiotics and host defenses leads to recurrent infections [10]. A microbial biofilm is a community composed of aggregated, organized, and functional microbial cells that are re-encapsulated in a matrix of extracellular polymeric substances, enabling them to adhere irreversibly to biotic and abiotic surfaces [11]. Therefore, biofilm control is a global challenge that requires the development of new bioactive compounds as alternatives to antibiotics and chemically synthesized substances [12].

Biosurfactants are emulsifier molecules that make surfaces more efficient and reduce the liquid interface tension [13]. Several microorganisms produce them. Biosurfactant-producing microorganisms live in fresh and saltwater, soil, sediment, and sludge [14]. Indeed, different classes of biosurfactants are produced by different strains, such as rhamnolipids, which are produced by *Pseudomonas aeruginosa* and *P. chlororaphis* [15], both of which are produced by *Candida bombicola* [16]; *Acinetobacter* sp. produces phospholipids [17]; lipopeptides are produced by *Bacillus subtilis* [18]; surfactin and iturin are produced by *Bacillus subtilis* and *B. amyloliquefaciens* [19]. *Bacillus* sp. produce a large range of lipopeptide biosurfactants that consist of a hydrophobic fatty acid linked to a hydrophilic peptide chain of seven or ten amino acids. *Bacillus subtilis* biosurfactin, an alipoheptapeptide that exhibits high emulsifying, antimicrobial, antiviral, and antitumor properties, is among the most effective biosurfactants [20]. The criterion for classification as a biosurfactant producer is the ability to reduce the surface tension of a solution to <40 mN/m. Critical micellar concentration (CMC) was used to assess the effectiveness of the surfactant [21]. In a study by Cheng et al. (2013), *Bacillus subtilis* TU2 was found to produce a biosurfactant with a CMC of 50 mg/L, which reduced the surface tension of water from 73 to 32.5 mN/M1 [22]. Furthermore, biosurfactants can increase the surface area and bioavailability of hydrophobic, water-insoluble substrates, bind to heavy metals, reduce and control biofilms, and detect quorum [23,24]. In addition, they possess antibacterial, anti-stick, and anti-biofilm properties against several bacterial strains, including those resistant to drugs, such as *Acinetobacter baumannii*, *Escherichia coli*, and methicillin-resistant *S. aureus* (MRSA) [24,25].

With advancements in science and technology, dosage forms have evolved from simple mixtures to highly sophisticated forms known as nanoemulsions. With nanotechnology, drug-loading capacity has been enhanced and dosage sizes have been reduced [26]. An emulsifying agent can produce nanoemulsions through dispersing one liquid (inner phase) into another (outer phase). They must be synthesized under tightly controlled conditions to prevent their physicochemical properties from being affected by their qualitative and quantitative compositions [27]. A recent study on the performance of surfactin and its variant fatty acyl glutamic acid-containing cinnamaldehyde, cinnamaldehyde nanoemulsions, and emulsions showed enhanced inhibition of bacterial growth against two common foodborne pathogens, *Escherichia coli* O157:H7 and *Listeria monocytogenes*, at concentrations lower than those of non-emulsified cinnamaldehyde, with greater inhibition from nanoemulsions [28]. In this study, we investigated the possible use of biosurfactants produced by a potential probiotic Bacillus strain as agents to control clinical pathogenic bacteria associated with aerobic vaginal infection. After the extraction of the biosurfactant, we developed a nanotechnology-based drug delivery system in the form of a nanoemulsion. Furthermore, the antibacterial and antibiofilm effects of cell-bound crude biosurfactant/biosurfactant in nanoemulsions was evaluated against a variety of biofilm-forming pathogens associated with aerobic vaginal infection.

## 2. Materials and Methods

### 2.1. Bacterial Strains and Growth Conditions

The bacterial strains used in this study were *E. coli* (MZ474969) and *S. aureus* (MZ475010 and MZ475016). To determine the inhibitory potential of crude biosurfactants from *Bacillus* sp. (HM117830), the pathogenic bacterial strains were cultured in Luria-Bertani (LB) broth (Difco) at 37 °C for 24 h. All selected strains were involved in vaginal infections. The potential probiotic strain *Bacillus* sp. (HM117830) was used to produce a biosurfactant, which was isolated from a hypersaline environment and previously investigated for its probiotic properties [29].

### 2.2. Production and Extraction of Biosurfactant

The production of biosurfactants by the *Bacillus* sp. strain was carried out by means of growing bacterial cells in minimal medium containing sterile olive oil (4%) as a source of carbon. To produce crude biosurfactant, an overnight culture of *Bacillus* sp. HM117834 (1%) was inoculated into 200 mL minimal medium and incubated at 37 °C for 48 h with shaking at 180 rpm. The culture medium was then centrifuged (4800 rpm for 20 min at 4 °C) to harvest the cells and avoid the denaturation of any existing protein compounds. The supernatant was then recovered and acidified with a 0.4% HCl solution at pH2 to precipitate the biosurfactant. Centrifugation was then performed to remove the bacteria, and the supernatant was collected after sterilizing filtration using a 0.22 µm filter. The mixture was stirred vigorously in a separatory funnel and allowed to stand for 5 min to separate the organic phase (the solvent containing the biosurfactant) from the aqueous phase. The organic phase was then dried via rotary evaporation under vacuum using a rotary evaporator (Heidolph) at 40 °C to remove the ethyl acetate and recover the crude biosurfactant. The biosurfactant production ability of *Bacillus* sp. was tested using an oil displacement test [24].

### 2.3. Nanoemulsion Preparation

The nanoemulsion was formulated based on a high-mechanical-energy emulsification method. A 10 wt% oily phase and 90 wt% aqueous phase were homogenized to form oil-in-water nanoemulsions. Two batches of nanoemulsions were performed with the following concentrations of biosurfactant: 1% and 3.33 wt% (BNE1% and BNE3.33%). The oily phase contained crude biosurfactant and oily vehicles (Isopropyl Myristate). The aqueous phase contained the aqueous vehicle (distilled water) and co-surfactant Tween 80. Preliminary studies were conducted, and the history of the research team in this field was used to determine the exact amounts of excipients in the formulations. A KINEMATIKA homogenizer (POLYTRON, Lucerne, Switzerland) was used for high-pressure homogenization. The aqueous and oil phases were performed at 13,000 rpm with a small inlet, resulting in extremely fine emulsions with extremely small droplets [30]. The obtained nanoemulsions were maintained at room temperature for 24 h before characterization.

### 2.4. Nanoemulsion Stability Testing

To assess the physical stability of the different nanoemulsions, screw-cap glass tubes containing approximately 5 g of the formulation were exposed to six series of cooling and heating cycles between 4 °C and 45 °C for 48 h in a heating–cooling incubator [31]. Formulations found to be stable at the temperatures used were additionally evaluated using a centrifugation assay (3500 rpm for 30 min). Formulations that did not exhibit any type of phase separation after the centrifugation test were evaluated via freeze–thaw stress tests using three series of cycles between 21 °C and 25 °C for 48 h [32]. The formulations were subsequently assessed for phase separation and drug precipitation [33]. Based on this stability study, only stable formulations were selected for further study.

### 2.5. Determination of Particle Size

The formulations were diluted with water to a concentration of 1% (*v*/*v*) in conical flasks and gently mixed. The droplet size distribution of the nanoemulsions was determined at 25 °C using a Zetasizer Nano-S dynamic light scattering particle size analyzer (Malvern Instruments, Malvern, UK). All experiments were performed in triplicate.

### 2.6. Determination of Zeta Potential

The electrophoretic mobility of the nanoemulsion formulation was measured using a Malvern Zetasizer Nano Z instrument (Malvern Instruments Ltd., Malvern, UK). Zeta potential was obtained using the Smoluchowski equation. The nanoemulsions were diluted with deionized water to a concentration of 1% (*v*/*v*) and gently mixed before measurement. Each sample was analyzed in triplicate using automatic mode.

### 2.7. Antibacterial Activity Study

#### 2.7.1. Antimicrobial Susceptibility Assay

Using the disk agar diffusion technique, the pathogenic strains were tested for susceptibility to different antimicrobials following CA SFM/EUCAST 2021. The antimicrobial disks that were used are listed in Table 1. The results of this assay were recorded at 18–24 h after incubation at 37 °C.

#### 2.7.2. Determination of Minimum Inhibitory Concentration (MIC) and Minimum Bactericidal Concentration (MBC)

The MICs of 1% nanoemulsion biosurfactant, 3.33% nanoemulsion biosurfactant, and crude biosurfactant 5% developed from *Bacillus* sp. were determined using a microdilution method [34]. Briefly, the samples were inoculated into 3 mL of MH broth and incubated overnight at 37 °C with shaking. The test solutions were placed in the wells of a 96-well plate (200 μL each). Then, 20 μL of the bacterial suspension with a final optical density (OD) of 0.01 at a wavelength of 600 nm (Infinite F200 PRO, TECAN, Lyon, France) and 10 μL of resazurin were deposited in all the wells. After 24 h of incubation at 37 °C, the OD was measured at 600 nm using an ELISA reader (Infinite F200 PRO, TECAN). The MIC value was determined as the concentration at which bacterial growth was inhibited. The negative control consisted only of media, whereas the positive control consisted only of inoculated bacteria without test products.

Another plate was prepared to study the minimum inhibitory concentration (MIC) of the different biosurfactant products in the presence of antibiotics to which the bacteria were resistant. Through conducting this test, we will be able to investigate the synergistic effect of biosurfactants and certain antibiotics against which bacteria are resistant.

It was determined that the MBC was defined as the lowest concentration of antimicrobial capable of inactivating 99.99% or more of the bacteria present. Each experiment was performed in triplicate.

### 2.8. Antibiofilm Assay

The method described by Sandasi et al. (2010) was used to determine the effect of *Bacillus* sp. crude biosurfactant and nanoemulsions on adherence to biofilm formation [35]. Bacterial cell cultures (20 µL) of each tested strain (10^6^ CFU/mL) and 100 µL of *Bacillus* sp. crude biosurfactant and nanoemulsions at MIC/2 supplemented with 2% glucose (*w*/*v*) in 96-well microtiter plates (Nunc, Roskilde, Denmark) were incubated for 24 h at 37 °C. After incubation, planktonic cells were removed, and the wells were washed with PBS (200 µL). After washing, the adherent cells were stained with 0.1% crystal violet for 30 min at 37 °C to visualize the biofilms formed by the test strains. Excess crystal violet dye was washed with PBS, and the plates were fixed using 95% ethanol (200 µL) and incubated for 15 min at 37 °C. Absorbance was measured at a wavelength of 570 nm. The results are expressed as a percentage of biofilm inhibition (BI): BI = [(OD negative control − OD Experimental)/OD negative control] × 100.

#### 2.8.1. Cell Surface Hydrophobicity

According to Bellon et al. [36], a solvent microbial adhesion test (MATS) was used to compare the hydrophobicity of *S. aureus* and *E. coli* before and after treatment with crude biosurfactant and different nanoemulsion-based biosurfactants. An experiment was conducted to evaluate the affinity of cells for a polar solvent (hexadecane). A final optical density of 0.3 at 600 nm was obtained via centrifuging bacterial cells at 16,128× *g* for 5 min at 4 °C and resuspending them in buffered saline (pH 7.0). Biosurfactant formulations were tested at a concentration of CMI/2 to prevent bactericidal activity. After incubation with BS for 6 h at 37 °C, the cell surface hydrophobicity percentage was determined via adding hexadecane to bacterial cultures in nutritional broth. A comparison of the samples before and after hexadecane extraction yielded hydrophobicity indices [37].

#### 2.8.2. Bacterial Cell Membrane Disintegration Test

The objective of this test was to evaluate the ability of the nanoemulsions and crude biosurfactants to destroy bacterial cells. Minimal medium was used for the preparation of bacterial suspensions. After 24 h of incubation at 37 °C, the cells were suspended in PBS (pH 7.4) after two washes. Bacteria were treated with different formulations at their MIC and incubated at 37 °C for 1 h. The absorbance of the supernatant at 260 nm was measured after the incubation period to examine the presence of any material released from the cell that absorbs ultraviolet light [38]. All experiments were performed in triplicates. The presence of materials that absorb at 260 nm indicates the presence of proteins detached from the bacterial cell and, consequently, the disintegration of the bacterial cell membrane.

#### 2.8.3. Statistical Analysis

Analysis of variance was used to compare data from each experiment, along with Kruskal–Wallis’s test for multiple comparisons of means (IBM SPSS Statistics V.24).

## 3. Results

### 3.1. Screening of Biosurfactant Production by Bacillus sp. and Evaluation of Nanoemulsion Properties

An oil-spreading assay was conducted to detect the presence of biosurfactants. According to this assay, the amount of oil displaced was directly proportional to the surfactant concentration. Oil-spreading assays using *Bacillus* sp. cell-free biosurfactant solutions were conducted based on the diameter and time of positive results (Table 2).

**Figure 1:**
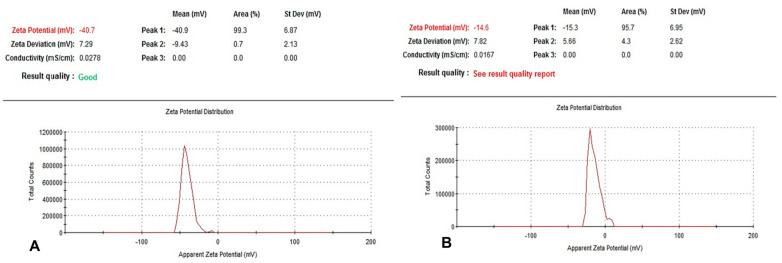
Zeta potential of BNE1% and BNE3.33%. The nanoparticles were negatively charged, and the zeta potential was −40 mV ± 7.29 mV and −14 mV ± 7.82 mV (**A**,**B**, respectively). (**A**) Zeta potential of BNE1%. (**B**) Zeta potential of BNE3.33%. The zeta potential of the biosurfactant-crosslinked nanoparticles decreased when the biosurfactant concentration was increased.

The nanoparticle sizes of BNE1% and BNE3.33% were around 133.8 nm and 226 nm (Table 2), respectively, confirming that these formulations were successfully located in the nanoscale range. Polydispersity indices were 0.362 and 0.332. The nanoparticles exhibited good dispersion. We found that BNE1% and BNE3.33% had zeta potentials values of −40 mV and −14 mV, respectively (Figure 1). Stability was confirmed by the lack of signs of surface modification and the appearance of the nanoemulsion, which is indicative of their stability.

### 3.2. Study of Antibacterial Activity

As shown in Table 3, the pathogenic strain *E. coli* MZ474969 was resistant to penicillin and nalidixic acid, but sensitive to gentamicin, Levofloxacin, and Norfloxacin. Antibiograms also prove that the pathogenic strains *S. aureus* MZ475010 and MZ475016 have significant resistance to antibiotics belonging to the family of aminosides represented by gentamicin and kanamycin, as well as to antibiotics belonging to the beta-lactam family, such as oxacillin and erythromycin, an antibiotic belonging to the macrolide family.

### 3.3. Antibacterial Activity of the Bacillus sp. Crude Biosurfactant and Nanoemulsions

The antibacterial potency of *Bacillus* sp. crude biosurfactants and nanoemulsions was evaluated through assessing the minimum inhibitory concentration (MIC) and minimum bactericidal concentration against the test pathogens. The MIC values of *Bacillus* sp. crude biosurfactant ranged from 4 to 2 mg/mL, and the MBC values were found to be two times higher than the MIC values (Table 4). In turn, the values of BNE1% and BNE3.33% MIC ranged from 2.5 to 1.25 mg/mL and from 0.26 to 0.13 mg/mL, respectively. However, the MBC values ranged from 5 to 2.5 mg/mL for BNE1% and 0.5 mg/mL for BNE3.33%.

The different formulations of biosurfactant in combination with antibiotics, such as ampicillin and oxacillin, led to an increase in the sensitivity of all strains toward antibiotic-resistant strains. BNE1% and 3.33% remained more effective at lower concentrations than crude biosurfactant did (Table 4).

### 3.4. Antibiofilm Potential of the Bacillus sp. Crude Biosurfactant and Nanoemulsions

**Figure 2:**
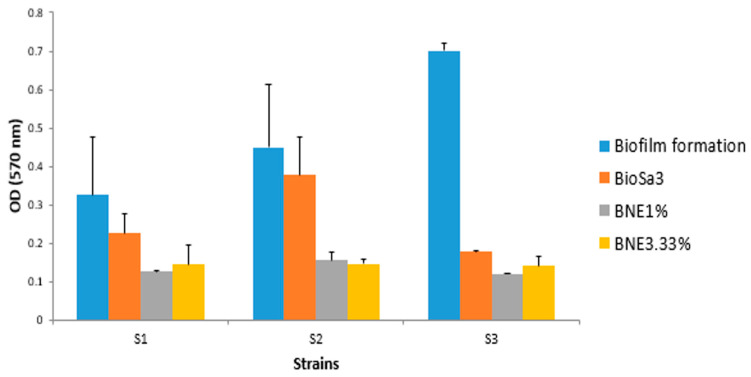
Comparative results of the study of the anti-adhesive effect of different formulations based on the biosurfactant against different strains. S1—*E. coli* MZ474969; S2—*S. aureus* MZ475010; S3—*S. aureus* MZ475016. BS—crude biosurfactant; BNE1%—1% nanoemulsion biosurfactant; BNE3.33%—3.33% nanoemulsion biosurfactant.

The antibiofilm potential of *Bacillus* sp. crude biosurfactants and nanoemulsions was determined according to their ability to alter the preformed biofilms of the pathogenic strains and inhibit their adhesion to the surface. Our results showed a pronounced antibiofilm effect of the biosurfactant on all the bacteria. This effect was enhanced with nanoemulsions compared to crude biosurfactants. Indeed, for *S. aureus* MZ475010, the optical density was reduced from 0.45 to 0.3 following treatment with crude biosurfactant. This decrease was accentuated following treatments with BNE1% (OD570 nm = 0.12) and BNE3.33% (OD570 nm = 0.19). Similarly, for the *E. coli* strain MZ474969, the two BNEs showed a much-improved antibiofilm effect compared to that exerted by the crude biosurfactant (Figure 2).

### 3.5. Effect of Bacillus sp. Crude Biosurfactant and Nanoemulsions on Cell Surface Hydrophobicity

**Figure 3:**
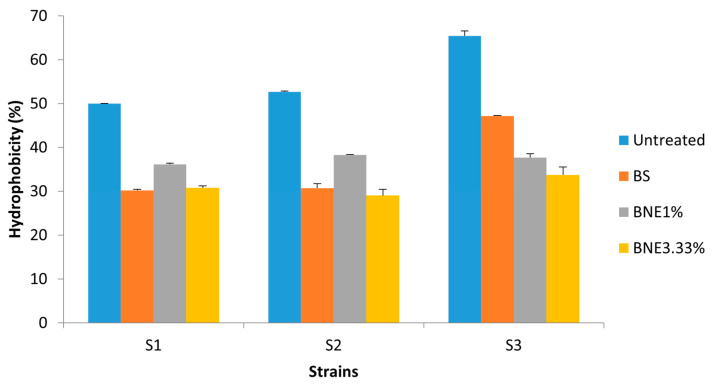
The affinity of treated and untreated bacterial cells with crude biosurfactant and nanoemulsions to hexadecane was used in the MATS test. S1—*E. coli* MZ474969; S2—*S. aureus* MZ475010; S3—*S. aureus* MZ475016. BS—crude biosurfactant; BNE1%—1% nanoemulsion biosurfactant; BNE3.33%—3.33% nanoemulsion biosurfactant.

After treatment of the strains tested with the various formulations based on biosurfactants, the results showed a decrease in the percentage of hydrophobicity for certain strains that changed character and became hydrophilic. Indeed, in the *S. aureus* MZ475016 strain, the percentage of hydrophobicity was approximately 60% (highly hydrophobic) and 20% (hydrophilic) following treatment (Figure 3). Compared with the crude biosurfactant, the nanoemulsions demonstrated a greater effect, of which BNE1% sometimes showed a greater effect than BNE3.33% in most strains.

### 3.6. Effect of Bacillus sp. Crude Biosurfactants and Nanoemulsions on Bacterial Cell Membrane Disintegration

**Figure 4:**
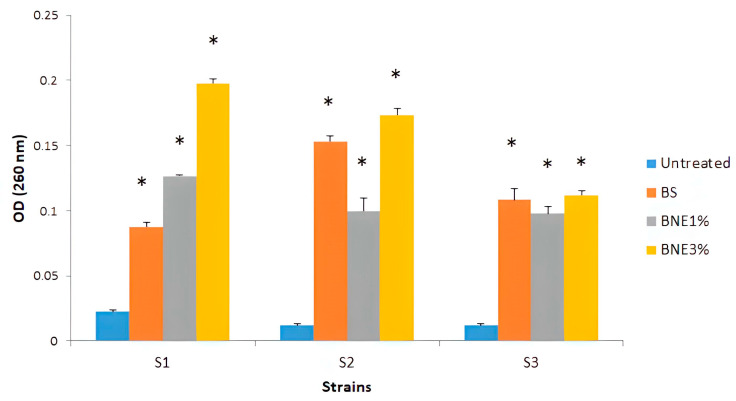
Effect of *Bacillus* sp. crude biosurfactants and nanoemulsions on bacterial cell membrane disintegration. S1—*E. coli* MZ474969; S2—*S. aureus* MZ475010; S3—*S. aureus* MZ475016. BS—crude biosurfactant; BNE1%—1% nanoemulsion biosurfactant; BNE3.33% nanoemulsion biosurfactant. Asterisks (*) indicate a significant difference in the same line between the treated and non-treated cells according to Kruskal–Wallis’s test (*p* < 0.05).

To explore the possibility that *Bacillus sp.* crude biosurfactant and nanoemulsions could have cell membrane disintegration capability in the pathogens tested, the absorbance of the supernatant at 260 nm was measured after treatment with different formulations of biosurfactant to examine the presence of any material released from the cell that absorbs ultraviolet light. Our results show that treatment with crude biosurfactants and nanoemulsions can affect cell membrane disintegration in tested pathogens. A significant difference was observed (*p* < 0.05) compared with the untreated strains. This was confirmed by the presence of UV-absorbing cellular products in the supernatant after treatment. The OD (260 nm) was around 0.01 to 0.04 for the untreated strains. After treatment with different formulations, OD260 nm increased to values ranging from 0.08 to 0.2 (Figure 4).

## 4. Discussion

Nanotechnology has played an important role in a range of scientific fields including physics, chemistry, engineering, medicine, and pharmaceuticals. Nanotechnology has numerous benefits in various fields of science. The application of nanotechnology in medicine for the prevention, diagnosis, and treatment of a wide range of illnesses has made nanotechnology an important field [11,39]. Several methods have been developed using nanotechnology to prevent, monitor, control, and cure diseases, including the combination of materials or devices with drugs or biomolecules, adding features such as slow and controlled drug release, increased tissue penetration efficiency, and protection against drug degradation [39]. Currently, liposomes, microemulsions, nanoemulsions, cyclodextrins, solid lipid nanoparticles, polymeric nanoparticles, and metallic nanoparticles are the most widely used nanosystems for delivery of bioactive substances [11]. The use of nanostructured systems for the treatment of infectious diseases that are resistant to conventional treatments such as antimicrobial drugs has become a promising field of research in recent years. As a result, patients with these diseases have improved their quality of life and life expectancy [40].

Molecular analysis suggests that the antimicrobial activity and mechanism of nanoemulsions occur through the ability of nanoemulsions to fuse with the outer membranes of microorganisms. Electrostatic interactions between the cationic charge of nanoparticles and the anionic charge of microorganisms [41] ultimately lead to the disruption of membrane bilayers and cellular permeability, which is the cause of their broad-spectrum activity [42]. It is important for nanoparticle stability in suspension as well as the initial adsorption of nanoparticles on cell membranes that the zeta potential is dependent on the surface charge (Hunter). In the endocytosis pathway, the uptake rate depends on particle size after adsorption [43]. Indeed, the control of the size and zeta potential of nanoparticles is thus critical for their efficacy in medication delivery [44,45]. According to Danaei et al. [46], particle sizes between 10 nm and 20 nm may be used to describe how drugs detach from various body organs. Liposomes and nanoliposomes based on phospholipids can deliver drugs with PDIs as low as 0.3 and 40, indicating homogeneity within the phospholipid membrane vesicles. The physical stability of emulsions is governed by their zeta potential, with a higher value (positive or negative) denoting greater stability [47]. The zeta potential value of the biosurfactant nanoemulsion indicated that different formulations were stable (Table 2). In most studies, oils or flavors are encapsulated with microparticles or nanoparticles, which are studied for their physical stability and biological activity, but not for volatile contents that may change once encapsulated [48].

Conventional antimicrobial agents are gradually becoming less effective, owing to the development of resistance mechanisms and biofilms by these microorganisms. Biofilms are communities of organized and functional microorganisms that adhere irreversibly to biotic or abiotic surfaces owing to their embedded matrix of extracellular polymeric substances [11]. The treatment and eradication of microbial biofilms are complex because of their genetic and structural dynamics. It has been suggested that sessile cells provide favorable conditions for microbial viability, making them less susceptible to elimination than planktonic forms of the same microorganisms [49]. As a result, planktonic microbes can resist antimicrobial agents using mechanisms such as quorum sensing and several other processes such as enzyme production, the expression of efflux pumps, and natural mutations [50]. In addition, the rates of metabolism and growth are low for microorganisms that exist in the form of biofilms, and the extracellular polymeric substance matrix, which is hydrophilic and anionic in nature, acts as an adsorbent, thus reducing the amount of antimicrobial agent available for interaction with biofilm microbes where an adequate amount of the antimicrobial drug does not reach the host tissues, rendering the therapy ineffective [51,52]. Therefore, it is likely that resistance to several antimicrobial agents can also occur if biofilms represent a mixture of different types of microorganisms living in a single community and that individual resistance mechanisms contribute to the resistance of the community [53]. Similar to other biofilm infections, bacterial vaginosis biofilms protect against antibiotics [54]. In addition to the pathogenesis of bacterial vaginosis, vaginal biofilms are also important for recurrence and treatment failure. In fact, in vitro studies have shown that *Gardnerella vaginalis* biofilms are highly resistant to the protective mechanisms of normal vaginal microflora, including hydrogen peroxide and lactic acid lactobacilli [55], and are also more tolerant to antibiotics [56].

To be effective against infection, an antimicrobial agent must be administered at a sufficiently high concentration to inhibit the proliferation of pathogenic bacteria at the infection site. Therefore, it is crucial that antibiotics reach their target sites in their active forms to ensure efficient binding, which interferes with the function of the target. Unlike planktonic cells of the same species, bacteria in biofilms are highly resistant to the antimicrobial agents used against planktonic cells of the same species. Bacteria in biofilms may have higher minimum inhibitory concentrations (MICs) than those in planktonic forms [11]. Consequently, nanotechnology is becoming more widely used in therapeutic measures, especially for the eradication of biofilms and the treatment of multidrug-resistant bacterial infections [57]. Although these systems have varying applications in the treatment of biofilms, nanotechnology-based drug delivery systems can facilitate drugs that interact directly with biofilm structures and act throughout the different stages of biofilm formation [11].

This study investigated the resistance of *E. coli* and *S. aureus* strains to certain antibiotics. Despite its resistance to penicillin and nalidixic acid, *E. coli* MZ474969 was sensitive to gentamicin, Levofloxacin, and Norfloxacin. A significant amount of resistance is also found in the pathogenic strains *S. aureus* MZ475010 and MZ475016 to an antibiotics from the aminoside family, including gentamicin and kanamycin. Additionally, erythromycin, an antibiotic belonging to the macrolide family, and antibiotics from the beta-lactam family, have been reported to be ineffective against these pathogens (Table 3). There is no doubt that the most significant multidrug-resistant bacteria include *S. aureus* that is resistant to methicillin, *Enterococci* that is resistant to vancomycin, and Gram-negative rods that produce extended-spectrum-lactamases or carbapenemases (such as *E. coli*, *Klebsiella pneumoniae*, *Acinetobacter baumannii*, and *Pseudomonas aeruginosa*) [58]. We evaluated the antibacterial potency of *Bacillus* sp. crude bio-surfactants and nanoemulsions through assessing the minimum inhibitory concentrations (MIC) and minimum bactericidal concentrations. *Bacillus* sp. crude biosurfactant had MIC values ranging from 4 to 2 mg/mL, and the MBC values were two times higher than the MIC values (Table 4). Accordingly, the BNE1% and BNE3.33% MIC values ranged from 2.5 to 1.25 mg/mL and 0.26 to 0.13 mg/mL, respectively. MBC values for BNE1% and BNE3.33% were 5 mg/mL–2.5 mg/mL, and 0.5 mg/mL, respectively. In comparison to crude biosurfactants, BNE1% and 3.33% were still more efficient at lower concentrations. Similarly, cinnamaldehyde nanoemulsions and emulsions showed higher inhibition of bacterial growth at concentrations lower than the MICs compared to non-emulsified cinnamaldehyde, with considerable inhibition from nanoemulsions [28]. Moreover, combining biosurfactants with antibiotics such as ampicillin and oxacillin increased the sensitivity of all strains to antibiotics (Table 4). To identify the most appropriate nanoemulsion preparation, Huwang et al. (2013) tested five nanoemulsion preparations against four *Acinetobacter baumannii* isolates. Among the formulations tested, a mixture of Triton X-100, soybean oil, and cetylpyridinium chloride showed the highest efficacy against *A. baumannii*, planktonic bacteria, and biofilm formation. Nanoemulsions have been shown to kill planktonic forms of the pathogen strain through their 1% CPC content but break down biofilms through their emulsified oil and detergent components [59]. Antimicrobial nanoemulsions (particle size, 100–800 nm) are effective against various microorganisms [60,61] at safe concentrations in the skin or mucous membranes of animals [62]. The outer membrane of microorganisms’ fuses with nanoemulsions to provide antimicrobial activity with an electrostatic interaction between the cationic nanoparticles and anionic microorganisms [41], which eventually disrupts the lipid bilayers of the membrane and its cellular components. Compared with other anti-adhesive agents, biosurfactants alter hydrophobicity, which decreases microbial adhesion to the surface of biofilms [24]. As a result, biosurfactants help form a stable biofilm because foreign sources such as microorganisms, hydrophobicity, or electrical charges cannot adhere to it. This property can be observed, for instance, in the surfactant produced by *Streptococcus thermophilus*, which prevents the accumulation of other thermophilic strains on steel surfaces [63]. According to a similar study, a biosurfactant produced from *Pseudomonas fluorescens* prevents *Listeria monocytogenes* from adhering to steel surfaces [64]. To test for the presence of any material that absorbs the UV light released from the cell, we measured the optical density at 260 nm after treatment with different formulations of the biosurfactant, as shown in Figure 4. The optical density values showed cell membrane disintegration in the tested pathogens of *E. coli* and *S. aureus* suspensions treated with nanoemulsions. As a result, *E. coli* and *S. aureus* cell membranes disintegrated, supporting our earlier inhibitory results. The amount of leakage depends on the increase in the concentration of eugenol nanoemulsions, which may reduce the hydrophobicity of bacterial surfaces, thus damaging cell membranes and causing content release [65,66]. Another recent study examined the antibacterial and antibiofilm effects of essential oils such as Origanum glandulosum on multidrug-resistant clinical isolates. At sub-MICs, both formulations demonstrated comparatively considerable efficacy against biofilm formation, with nanoemulsions being more effective than nanocapsules. These results indicate that essential oil nanoformulations are highly recommended for use in therapy as antibiotic alternatives [48].

Numerous innovative approaches for enhancing traditional antibiotic therapy and thwarting the advent of multidrug resistance have been made possible through the application of nanotechnology. Nanoemulsions containing biosurfactants have a considerable impact on biofilm development, pathogen growth, and hydrophobicity, creating an appealing alternative to dramatically reduce the negative effects of biofilm formation on human health.

## 5. Conclusions

In the search for alternative therapies to treat and control microbial diseases associated with biofilms and antibiotic resistance, it appears that the pursuit of innovative approaches to improving human life will continue. Based on several findings, it may be inferred that nanotechnology, owing to its vast potential in drug delivery systems, may be an effective alternative for the treatment of microbial biofilms. This study demonstrated that the biosurfactant in nanoemulsions has an antibiofilm effect against clinical pathogens. Owing to the many physiological barriers involved after exposure to nanoparticles, further investigation is needed to establish adequate in vivo models. In the near future, investing in nano-delivery systems may be the best way to combat multidrug resistance in bacteria.

## Figures and Tables

**Table 1:** List of antibiotics tested for the different strains studied.

Strain	*S. aureus*		*E. coli*	
**Antibiotic family**	**Beta-lactams**	Penicillin G (1 UI) Oxacillin (5 µg)	**Beta-lactams**	Ampicillin (10 µg)
	**Aminosides**	Kanamycin (30 µg) Gentamicin (10 µg)	**Aminosides**	Gentamicin (10 µg)
	**Macrolides**	Erythromycin (15 µg)	**Fluoroquinolones**	Levofloxacin (5 µg) Norfloxacin (10 µg) Nalidixic acid (30 µg)

**Table 2:** Screening of biosurfactant production by *Bacillus* sp. HM117830 and evaluation of nanoemulsion properties.

Strain	Crude Biosurfactant	BNE1%			BNE3.3%		
	Oil-Spreading Test	Zeta Potential	Particle Size	Polydispersity Index	Zeta Potential	Particle Size	Polydispersity Index
***Bacillus* sp. HM117830**	Positive	−40 mV	133.8 nm	0.369	−14 mV	226 nm	0.332

**Table 3:** Antibiogram results for strains of *S. aureus* and *E. coli*.

Strain	Beta-Lactams			Aminosides		Macrolides	Fluoroquinolones		
	PG (1 UI)	O (5 µg)	A (10 µg)	K (30 µg)	G (10 µg)	E (15 µg)	L (5 µg)	N (10 µg)	Na (30 µg)
*E. coli* MZ474969	-	-	**R**	-	**S**	-	**S**	**S**	**R**
*S. aureus* MZ475010	**S**	**R**	-	**R**	**R**	**R**	-	-	-
*S. aureus* MZ475016	**R**	**R**	-	**R**	**R**	**R**	-	-	-

PG—Penicillin G; O—Oxacillin; A—Ampicillin; K—Kanamycin; G—Gentamicin; E—Erythromycin; L—Levofloxacin; N—Norfloxacin; Na—nalidixic acid; R—resistant; S—sensitive.

**Table 4:** Antibacterial activity of the *Bacillus* sp. crude biosurfactant and nanoemulsions.

Strain	*Bacillus* sp. Crude Biosurfactant (mg/mL)		BNE1% (mg/mL)		BNE3.33% (mg/mL)		In Addition of Antibiotics		
							*Bacillus* sp. Crude Biosurfactant (mg/mL)	BNE1% (mg/mL)	BNE3.33% (mg/mL)
	MIC	MBC	MIC	MBC	MIC	MBC	MIC	MIC	MIC
*E. coli* MZ474969	2	8	1.25	5	0.5	0.5	0.007	0.019	0.065
*S. aureus* MZ475010	4	4	1.25	2.5	0.26	0.5	0.007	0.039	0.13
*S. aureus* MZ475016	4	4	1.25	2.5	0.26	0.5	0.007	0.039	0.26

## Data Availability

The data presented in this study are available in this article.
